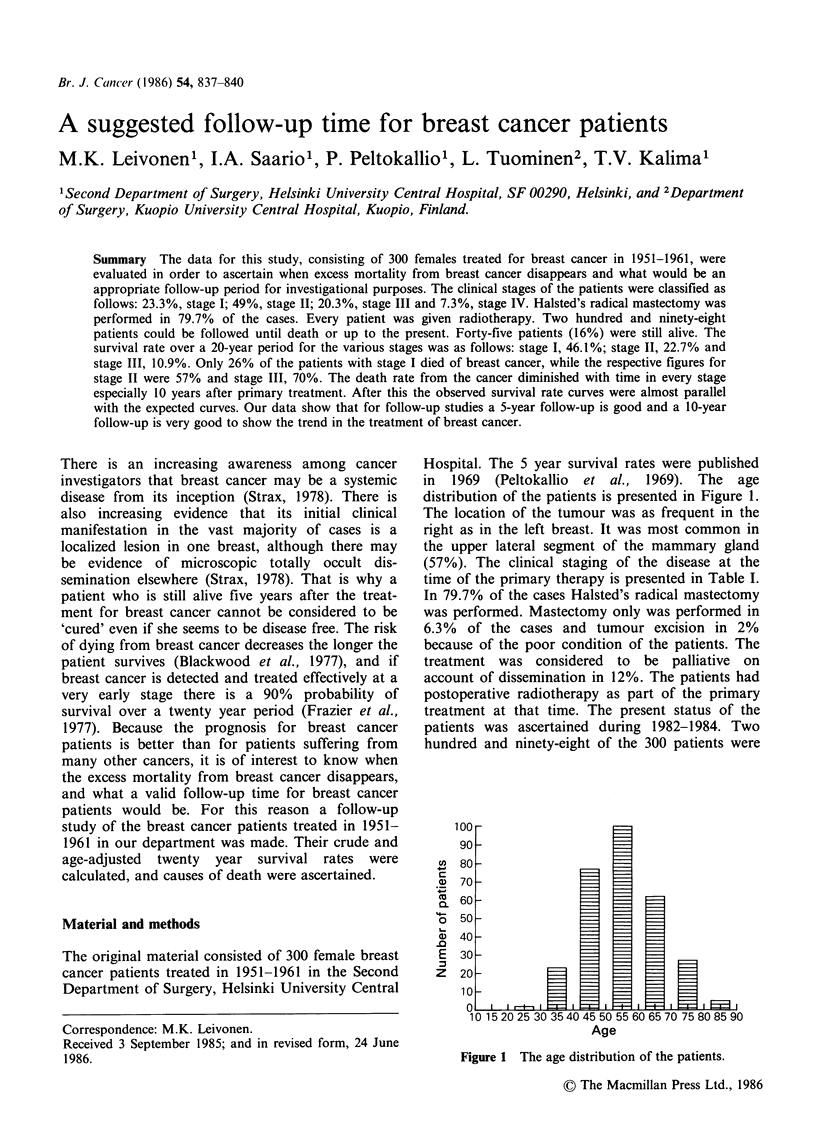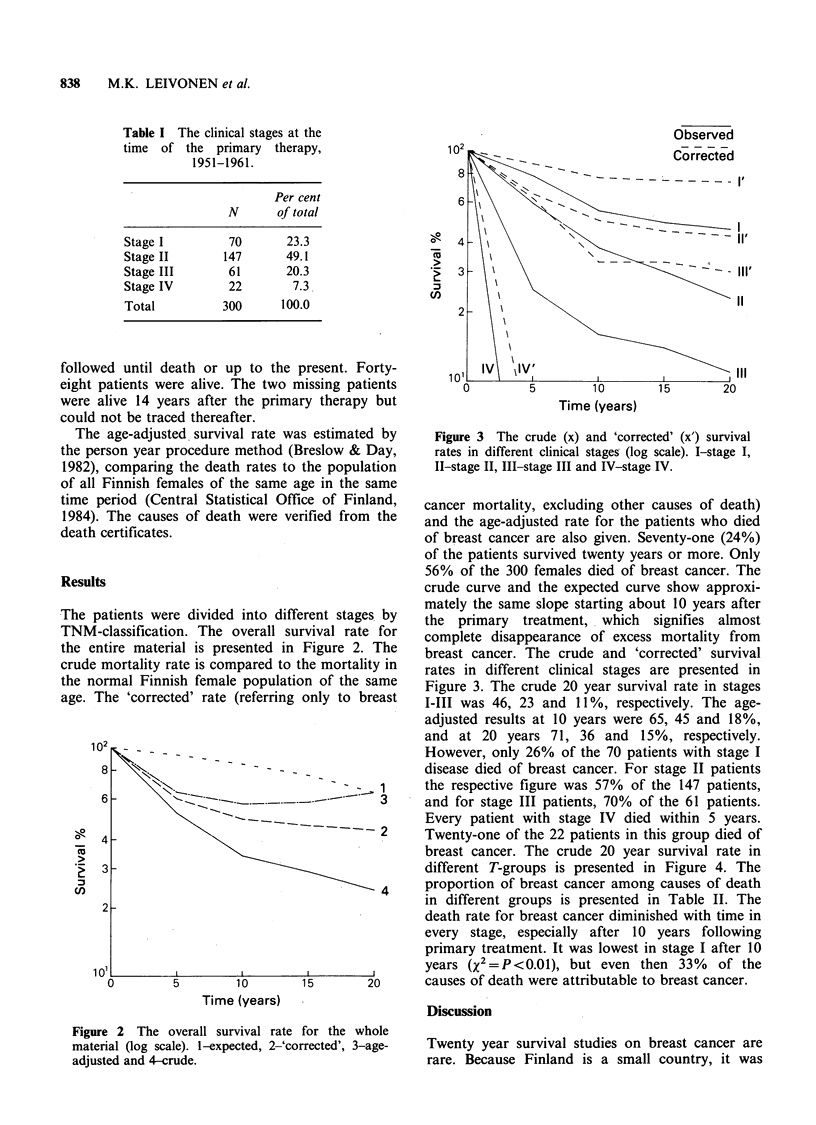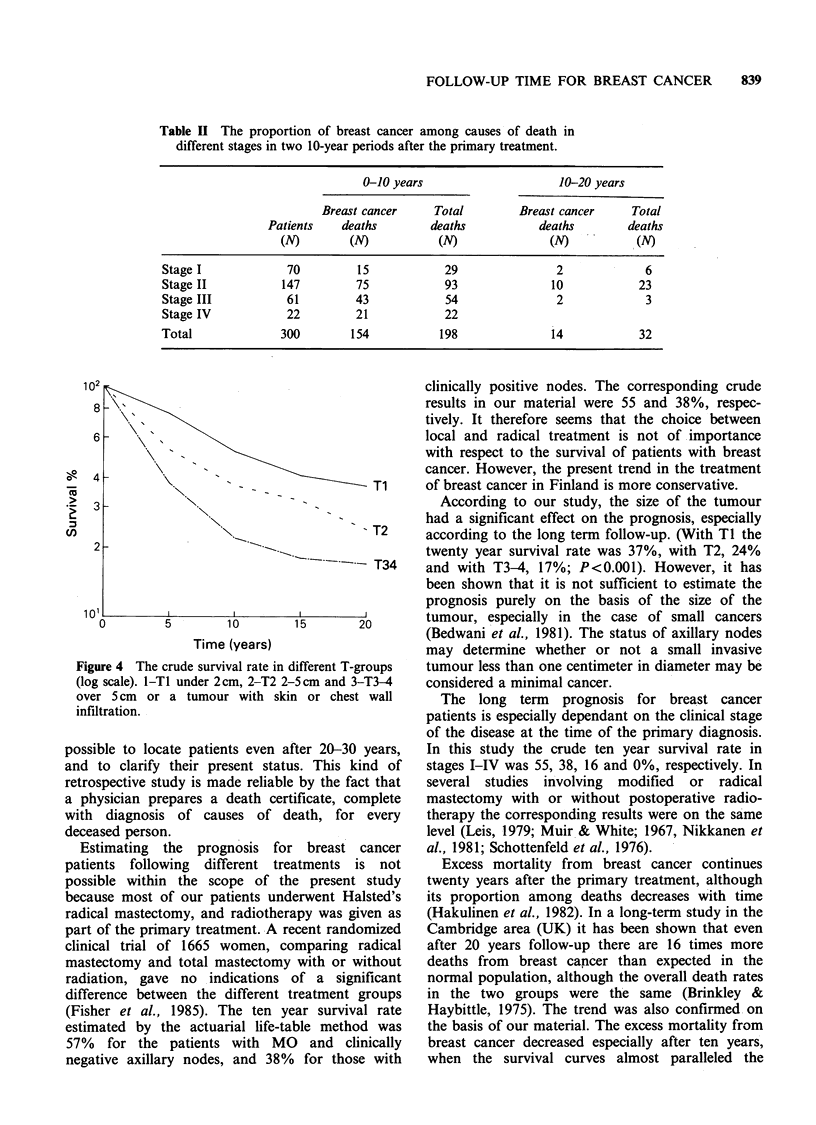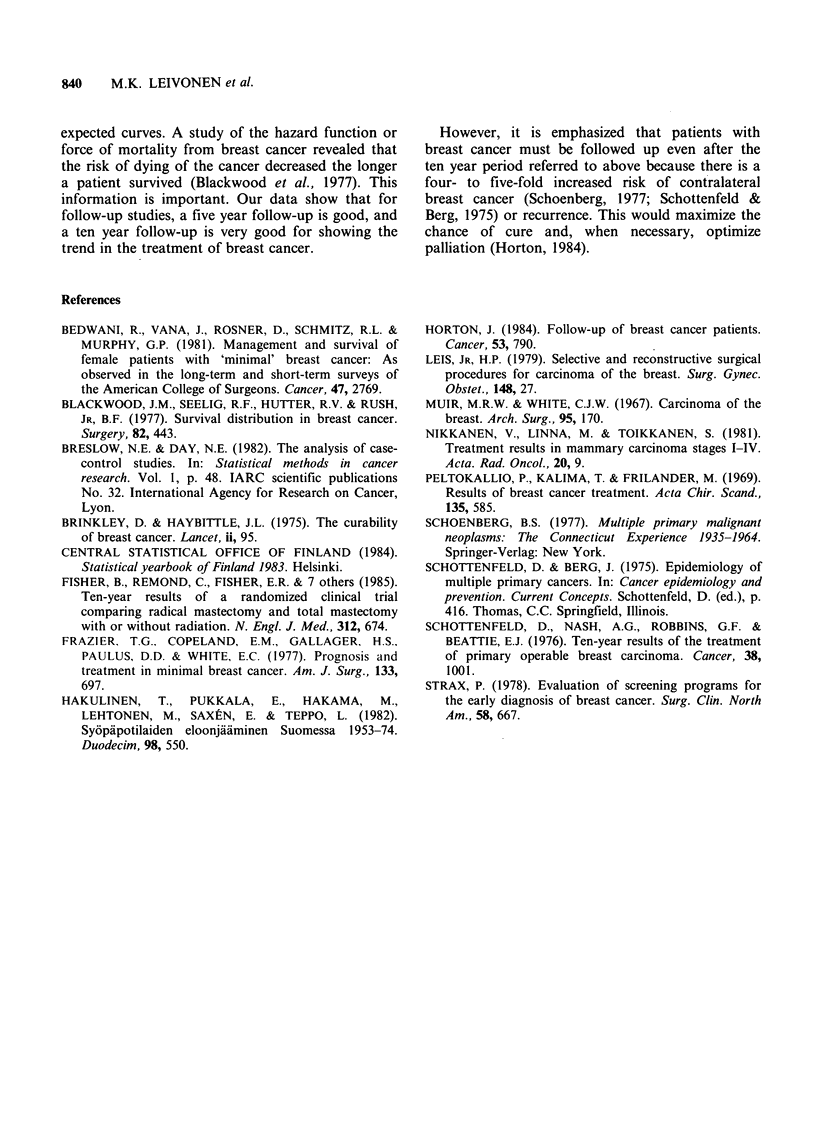# A suggested follow-up time for breast cancer patients.

**DOI:** 10.1038/bjc.1986.248

**Published:** 1986-11

**Authors:** M. K. Leivonen, I. A. Saario, P. Peltokallio, L. Tuominen, T. V. Kalima

## Abstract

The data for this study, consisting of 300 females treated for breast cancer in 1951-1961, were evaluated in order to ascertain when excess mortality from breast cancer disappears and what would be an appropriate follow-up period for investigational purposes. The clinical stages of the patients were classified as follows: 23.3%, stage I; 49%, stage II; 20.3%, stage III and 7.3%, stage IV. Halsted's radical mastectomy was performed in 79.7% of the cases. Every patient was given radiotherapy. Two hundred and ninety-eight patients could be followed until death or up to the present. Forty-five patients (16%) were still alive. The survival rate over a 20-year period for the various stages was as follows: stage I, 46.1%; stage II, 22.7% and stage III, 10.9%. Only 26% of the patients with stage I died of breast cancer, while the respective figures for stage II were 57% and stage III, 70%. The death rate from the cancer diminished with time in every stage especially 10 years after primary treatment. After this the observed survival rate curves were almost parallel with the expected curves. Our data show that for follow-up studies a 5-year follow-up is good and a 10-year follow-up is very good to show the trend in the treatment of breast cancer.


*Br. J. Cancer* (1986) **54**, 837–840

### A suggested follow-up time for breast cancer patients

M.K. Leivonen[1], I.A. Saario[1], P. Peltokallio[1], L. Tuominen[2], T.V. Kalima[1]

[1]*Second Department of Surgery, Helsinki University Central Hospital, SF 00290, Helsinki, and* [2]*Department of Surgery, Kuopio University Central Hospital, Kuopio, Finland.*

**Summary** The data for this study, consisting of 300 females treated for breast cancer in 1951–1961, were evaluated in order to ascertain when excess mortality from breast cancer disappears and what would be an appropriate follow-up period for investigational purposes. The clinical stages of the patients were classified as follows: 23.3%, stage I; 49%, stage II; 20.3%, stage III and 7.3%, stage IV. Halsted's radical mastectomy was performed in 79.7% of the cases. Every patient was given radiotherapy. Two hundred and ninety-eight patients could be followed until death or up to the present. Forty-five patients (16%) were still alive. The survival rate over a 20-year period for the various stages was as follows: stage I, 46.1%; stage II, 22.7% and stage III, 10.9%. Only 26% of the patients with stage I died of breast cancer, while the respective figures for stage II were 57% and stage III, 70%. The death rate from the cancer diminished with time in every stage especially 10 years after primary treatment. After this the observed survival rate curves were almost parallel with the expected curves. Our data show that for follow-up studies a 5-year follow-up is good and a 10-year follow-up is very good to show the trend in the treatment of breast cancer.

There is an increasing awareness among cancer investigators that breast cancer may be a systemic disease from its inception (Strax, 1978). There is also increasing evidence that its initial clinical manifestation in the vast majority of cases is a localized lesion in one breast, although there may be evidence of microscopic totally occult dissemination elsewhere (Strax, 1978). That is why a patient who is still alive five years after the treatment for breast cancer cannot be considered to be 'cured' even if she seems to be disease free. The risk of dying from breast cancer decreases the longer the patient survives (Blackwood *et al.*, 1977), and if breast cancer is detected and treated effectively at a very early stage there is a 90% probability of survival over a twenty year period (Frazier *et al.*, 1977). Because the prognosis for breast cancer patients is better than for patients suffering from many other cancers, it is of interest to know when the excess mortality from breast cancer disappears, and what a valid follow-up time for breast cancer patients would be. For this reason a follow-up study of the breast cancer patients treated in 1951–1961 in our department was made. Their crude and age-adjusted twenty year survival rates were calculated, and causes of death were ascertained.

### Material and methods

The original material consisted of 300 female breast cancer patients treated in 1951–1961 in the Second Department of Surgery, Helsinki University Central Hospital. The 5 year survival rates were published in 1969 (Peltokallio *et al.*, 1969). The age distribution of the patients is presented in Figure 1. The location of the tumour was as frequent in the right as in the left breast. It was most common in the upper lateral segment of the mammary gland (57%). The clinical staging of the disease at the time of the primary therapy is presented in Table I. In 79.7% of the cases Halsted's radical mastectomy was performed. Mastectomy only was performed in 6.3% of the cases and tumour excision in 2% because of the poor condition of the patients. The treatment was considered to be palliative on account of dissemination in 12%. The patients had postoperative radiotherapy as part of the primary treatment at that time. The present status of the patients was ascertained during 1982–1984. Two hundred and ninety-eight of the 300 patients were

**Figure 1** The age distribution of the patients.

Correspondence: M.K. Leivonen.
Received 3 September 1985; and in revised form, 24 June 1986.

© The Macmillan Press Ltd., 1986

**Table I** The clinical stages at the time of the primary therapy, 1951–1961.

| | *N* | *Per cent of total* |
|---|---|---|
| Stage I | 70 | 23.3 |
| Stage II | 147 | 49.1 |
| Stage III | 61 | 20.3 |
| Stage IV | 22 | 7.3 |
| Total | 300 | 100.0 |

followed until death or up to the present. Forty-eight patients were alive. The two missing patients were alive 14 years after the primary therapy but could not be traced thereafter.

The age-adjusted survival rate was estimated by the person year procedure method (Breslow & Day, 1982), comparing the death rates to the population of all Finnish females of the same age in the same time period (Central Statistical Office of Finland, 1984). The causes of death were verified from the death certificates.

### Results

The patients were divided into different stages by TNM-classification. The overall survival rate for the entire material is presented in Figure 2. The crude mortality rate is compared to the mortality in the normal Finnish female population of the same age. The 'corrected' rate (referring only to breast

**Figure 2** The overall survival rate for the whole material (log scale). 1–expected, 2–'corrected', 3–age-adjusted and 4–crude.

**Figure 3** The crude (x) and 'corrected' (x') survival rates in different clinical stages (log scale). I–stage I, II–stage II, III–stage III and IV–stage IV.

cancer mortality, excluding other causes of death) and the age-adjusted rate for the patients who died of breast cancer are also given. Seventy-one (24%) of the patients survived twenty years or more. Only 56% of the 300 females died of breast cancer. The crude curve and the expected curve show approximately the same slope starting about 10 years after the primary treatment, which signifies almost complete disappearance of excess mortality from breast cancer. The crude and 'corrected' survival rates in different clinical stages are presented in Figure 3. The crude 20 year survival rate in stages I-III was 46, 23 and 11%, respectively. The age-adjusted results at 10 years were 65, 45 and 18%, and at 20 years 71, 36 and 15%, respectively. However, only 26% of the 70 patients with stage I disease died of breast cancer. For stage II patients the respective figure was 57% of the 147 patients, and for stage III patients, 70% of the 61 patients. Every patient with stage IV died within 5 years. Twenty-one of the 22 patients in this group died of breast cancer. The crude 20 year survival rate in different *T*-groups is presented in Figure 4. The proportion of breast cancer among causes of death in different groups is presented in Table II. The death rate for breast cancer diminished with time in every stage, especially after 10 years following primary treatment. It was lowest in stage I after 10 years ($\chi^2 = P < 0.01$), but even then 33% of the causes of death were attributable to breast cancer.

### Discussion

Twenty year survival studies on breast cancer are rare. Because Finland is a small country, it was

**Table II** The proportion of breast cancer among causes of death in different stages in two 10-year periods after the primary treatment.

| | | *0–10 years* | | *10–20 years* | |
|---|---|---|---|---|---|
| | *Patients* (*N*) | *Breast cancer deaths* (*N*) | *Total deaths* (*N*) | *Breast cancer deaths* (*N*) | *Total deaths* (*N*) |
| Stage I | 70 | 15 | 29 | 2 | 6 |
| Stage II | 147 | 75 | 93 | 10 | 23 |
| Stage III | 61 | 43 | 54 | 2 | 3 |
| Stage IV | 22 | 21 | 22 | | |
| Total | 300 | 154 | 198 | 14 | 32 |

**Figure 4** The crude survival rate in different T-groups (log scale). 1–T1 under 2 cm, 2–T2 2–5 cm and 3–T3–4 over 5 cm or a tumour with skin or chest wall infiltration.

possible to locate patients even after 20–30 years, and to clarify their present status. This kind of retrospective study is made reliable by the fact that a physician prepares a death certificate, complete with diagnosis of causes of death, for every deceased person.

Estimating the prognosis for breast cancer patients following different treatments is not possible within the scope of the present study because most of our patients underwent Halsted's radical mastectomy, and radiotherapy was given as part of the primary treatment. A recent randomized clinical trial of 1665 women, comparing radical mastectomy and total mastectomy with or without radiation, gave no indications of a significant difference between the different treatment groups (Fisher *et al.*, 1985). The ten year survival rate estimated by the actuarial life-table method was 57% for the patients with MO and clinically negative axillary nodes, and 38% for those with clinically positive nodes. The corresponding crude results in our material were 55 and 38%, respectively. It therefore seems that the choice between local and radical treatment is not of importance with respect to the survival of patients with breast cancer. However, the present trend in the treatment of breast cancer in Finland is more conservative.

According to our study, the size of the tumour had a significant effect on the prognosis, especially according to the long term follow-up. (With T1 the twenty year survival rate was 37%, with T2, 24% and with T3–4, 17%; $P<0.001$). However, it has been shown that it is not sufficient to estimate the prognosis purely on the basis of the size of the tumour, especially in the case of small cancers (Bedwani *et al.*, 1981). The status of axillary nodes may determine whether or not a small invasive tumour less than one centimeter in diameter may be considered a minimal cancer.

The long term prognosis for breast cancer patients is especially dependant on the clinical stage of the disease at the time of the primary diagnosis. In this study the crude ten year survival rate in stages I–IV was 55, 38, 16 and 0%, respectively. In several studies involving modified or radical mastectomy with or without postoperative radiotherapy the corresponding results were on the same level (Leis, 1979; Muir & White; 1967, Nikkanen *et al.*, 1981; Schottenfeld *et al.*, 1976).

Excess mortality from breast cancer continues twenty years after the primary treatment, although its proportion among deaths decreases with time (Hakulinen *et al.*, 1982). In a long-term study in the Cambridge area (UK) it has been shown that even after 20 years follow-up there are 16 times more deaths from breast cancer than expected in the normal population, although the overall death rates in the two groups were the same (Brinkley & Haybittle, 1975). The trend was also confirmed on the basis of our material. The excess mortality from breast cancer decreased especially after ten years, when the survival curves almost paralleled the expected curves. A study of the hazard function or force of mortality from breast cancer revealed that the risk of dying of the cancer decreased the longer a patient survived (Blackwood *et al.*, 1977). This information is important. Our data show that for follow-up studies, a five year follow-up is good, and a ten year follow-up is very good for showing the trend in the treatment of breast cancer.

However, it is emphasized that patients with breast cancer must be followed up even after the ten year period referred to above because there is a four- to five-fold increased risk of contralateral breast cancer (Schoenberg, 1977; Schottenfeld & Berg, 1975) or recurrence. This would maximize the chance of cure and, when necessary, optimize palliation (Horton, 1984).

### References

BEDWANI, R., VANA, J., ROSNER, D., SCHMITZ, R.L. & MURPHY, G.P. (1981). Management and survival of female patients with 'minimal' breast cancer: As observed in the long-term and short-term surveys of the American College of Surgeons. *Cancer*, **47**, 2769.

BLACKWOOD, J.M., SEELIG, R.F., HUTTER, R.V. & RUSH, JR, B.F. (1977). Survival distribution in breast cancer. *Surgery*, **82**, 443.

BRESLOW, N.E. & DAY, N.E. (1982). The analysis of case-control studies. In: *Statistical methods in cancer research*. Vol. 1, p. 48. IARC scientific publications No. 32. International Agency for Research on Cancer, Lyon.

BRINKLEY, D. & HAYBITTLE, J.L. (1975). The curability of breast cancer. *Lancet*, **ii**, 95.

CENTRAL STATISTICAL OFFICE OF FINLAND (1984). *Statistical yearbook of Finland 1983*. Helsinki.

FISHER, B., REMOND, C., FISHER, E.R. & 7 others (1985). Ten-year results of a randomized clinical trial comparing radical mastectomy and total mastectomy with or without radiation. *N. Engl. J. Med.*, **312**, 674.

FRAZIER, T.G., COPELAND, E.M., GALLAGER, H.S., PAULUS, D.D. & WHITE, E.C. (1977). Prognosis and treatment in minimal breast cancer. *Am. J. Surg.*, **133**, 697.

HAKULINEN, T., PUKKALA, E., HAKAMA, M., LEHTONEN, M., SAXÉN, E. & TEPPO, L. (1982). Syöpäpotilaiden eloonjääminen Suomessa 1953–74. *Duodecim*, **98**, 550.

HORTON, J. (1984). Follow-up of breast cancer patients. *Cancer*, **53**, 790.

LEIS, JR, H.P. (1979). Selective and reconstructive surgical procedures for carcinoma of the breast. *Surg. Gynec. Obstet.*, **148**, 27.

MUIR, M.R.W. & WHITE, C.J.W. (1967). Carcinoma of the breast. *Arch. Surg.*, **95**, 170.

NIKKANEN, V., LINNA, M. & TOIKKANEN, S. (1981). Treatment results in mammary carcinoma stages I–IV. *Acta. Rad. Oncol.*, **20**, 9.

PELTOKALLIO, P., KALIMA, T. & FRILANDER, M. (1969). Results of breast cancer treatment. *Acta Chir. Scand.*, **135**, 585.

SCHOENBERG, B.S. (1977). *Multiple primary malignant neoplasms: The Connecticut Experience 1935–1964*. Springer-Verlag: New York.

SCHOTTENFELD, D. & BERG, J. (1975). Epidemiology of multiple primary cancers. In: *Cancer epidemiology and prevention. Current Concepts*. Schottenfeld, D. (ed.), p. 416. Thomas, C.C Springfield, Illinois.

SCHOTTENFELD, D., NASH, A.G., ROBBINS, G.F. & BEATTIE, E.J. (1976). Ten-year results of the treatment of primary operable breast carcinoma. *Cancer*, **38**, 1001.

STRAX, P. (1978). Evaluation of screening programs for the early diagnosis of breast cancer. *Surg. Clin. North Am.*, **58**, 667.